# Supplementary material for: A Critical View of the Application of the APEX Software (Aqueous Photochemistry of Environmentally-Occurring Xenobiotics) to Predict Photoreaction Kinetics in Surface Freshwaters
Source: Molecules. 2019 Dec 18;25(1):9. doi: 10.3390/molecules25010009 (PMC7017383; doi:10.3390/molecules25010009)
Supplement: Supplementary file 1 [file molecules-25-00009-s001.zip › 3.2.4_gcc-4.4.0.zip!/3.2.4_gcc-4.4.0/3.2.4_gcc-4.4.0/doc/octave/html/interpreter/Sparse-Matrices-in-Oct_002dFiles.html]

Sparse Matrices in Oct-Files - Untitled


Next: Accessing Global Variables in Oct-Files,
Previous: Structures in Oct-Files,
Up: Oct-Files


---

#### A.1.6 Sparse Matrices in Oct-Files

There are three classes of sparse objects that are of interest to the
user.

`SparseMatrix`: A double precision sparse matrix class `SparseComplexMatrix`: A complex sparse matrix class `SparseBoolMatrix`: A boolean sparse matrix class

All of these classes inherit from the `Sparse<T>` template class,
and so all have similar capabilities and usage. The `Sparse<T>`
class was based on Octave `Array<T>` class, and so users familiar
with Octave's `Array` classes will be comfortable with the use of
the sparse classes.

The sparse classes will not be entirely described in this section, due
to their similarity with the existing `Array` classes. However,
there are a few differences due the different nature of sparse objects,
and these will be described. Firstly, although it is fundamentally
possible to have N-dimensional sparse objects, the Octave sparse classes do
not allow them at this time. So all operations of the sparse classes
must be 2-dimensional. This means that in fact `SparseMatrix` is
similar to Octave's `Matrix` class rather than its
`NDArray` class.

- Array and Sparse Differences- Creating Sparse Matrices in Oct-Files- Using Sparse Matrices in Oct-Files
